# Quantitative Evaluation of *CFTR* Gene Expression: A Comparison between Relative Quantification by Real-Time PCR and Absolute Quantification by Droplet Digital PCR

**DOI:** 10.3390/genes14091781

**Published:** 2023-09-09

**Authors:** Sabina Maria Bruno, Giovanna Blaconà, Stefania Lo Cicero, Germana Castelli, Mariarita Virgulti, Giancarlo Testino, Silvia Pierandrei, Andrea Fuso, Giuseppe Cimino, Giampiero Ferraguti, Adriana Eramo, Marco Lucarelli

**Affiliations:** 1 Department of Experimental Medicine, Sapienza University of Rome, 00161 Rome, Italy; sabinamaria.bruno@gmail.com (S.M.B.); giovannablak@gmail.com (G.B.); virgulti.1949280@studenti.uniroma1.it (M.V.); gi.testino@tiscali.it (G.T.); pierandrei.silvia@gmail.com (S.P.); andrea.fuso@uniroma1.it (A.F.); giampiero.ferraguti@uniroma1.it (G.F.); 2 Department of Oncology and Molecular Medicine, National Institute of Health, Istituto Superiore di Sanità, ISS, 00161 Rome, Italy; stefania.locicero@iss.it (S.L.C.); germana.castelli@iss.it (G.C.); adriana.eramo@iss.it (A.E.); 3 Cystic Fibrosis Reference Center of Lazio Region, Policlinico Umberto I University Hospital, 00161 Rome, Italy; ciminolo@tiscali.it

**Keywords:** cystic fibrosis, *CFTR* expression, real-time PCR, digital droplet PCR

## Abstract

In the precision medicine era of cystic fibrosis (CF), therapeutic interventions, by the so-called modulators, target the cystic fibrosis transmembrane conductance regulator (CFTR) protein. The levels of targetable CFTR proteins are a main variable in the success of patient-specific therapy. In turn, the CFTR protein level depends, at least in part, on the level of *CFTR* mRNA. Many mechanisms can modulate the *CFTR* mRNA level, for example, transcriptional rate, stability of the mRNA, epigenetics, and pathogenic variants that can affect mRNA production and degradation. Independently from the causes of variable *CFTR* mRNA levels, their exact quantitative assessment is of great importance in CF. Methods with high analytical sensitivity, precision, and accuracy are mandatory for the quantitative evaluation aimed at the amelioration of the diagnostic, prognostic, and therapeutic aspects. This paper compares, for the first time, two *CFTR* gene expression quantification methods: a well-established method for the relative quantification of *CFTR* mRNA using a real-time PCR and an innovative method for its absolute quantification using a droplet digital PCR. No comprehensive methods for absolute *CFTR* quantification via droplet digital PCR have been published so far. The accurate quantification of *CFTR* expression at the mRNA level is a critical step for the personalized therapeutic approaches of CF.

## 1. Introduction

The cystic fibrosis transmembrane conductance regulator (*CFTR*) gene encodes for a transmembrane protein, which plays several cellular roles. The main role is that of the channel for chloride ion transport across the plasma membrane, ensuring the proper hydration of the surface of epithelia, which is mandatory for their correct functioning. A severely defective quantity or activity of the CFTR protein originates in cystic fibrosis (CF), targeting respiratory, pancreatic, and vas deferens epithelia. The possible pathological consequences are lung function impairment, pancreatic insufficiency, and male infertility. Respiratory insufficiency is the main cause of morbidity and mortality. The long way road from the gene to the properly located and active protein is studded with pitfalls. Several checks have to be passed from the mRNA transcription, mRNA maturation, translation, post-translational modification, and maturation processes within the endoplasmic reticulum and Golgi apparatus to plasma membrane positioning and functioning (with a half-life of about 12 to 24 h) [1,2,3].

The number of DNA variants of the *CFTR* gene is high (2114 in the CFTR1 database last accessed on 28 August 2023). Each production and maturation step can be affected by DNA variants. Although an ameliorated knowledge of the functional consequences of *CFTR* variants has been reached (the CFTR2 database was last accessed on 28 August 2023) [4], the characterization of the clinical consequences of the variants found by *CFTR* extensive mutational searches [5,6] is still a difficult task. On the other hand, these aspects have a great impact on the relationship between genotype and phenotype and on the diagnostic, prognostic, and therapeutic decisional processes [7,8,9,10,11,12,13,14]. In fact, the precision diagnostics applied at the *CFTR* gene level made the recent advent of CF precision therapy possible, which, in turn, requires a full assessment of the molecular mechanism(s) of *CFTR* pathogenic variants [15,16,17]. For some of the *CFTR* pathogenic variants, the so-called modulatory therapy is presently available, based on drugs acting on the CFTR protein at the biochemical level. They can enhance the folding of CFTR within the endoplasmic reticulum (correctors) or its activity when in the plasma membrane (potentiators), according to the specific functional defects of the CFTR protein.

The quantitative level of *CFTR* mRNA is crucial for the subsequent steps aimed at the production of the greatest quantity of functional (possibly after therapy with modulators) CFTR protein. The experimental assessment of the *CFTR* mRNA level, variable for physiologic interindividual differences and/or for the pathological effect of DNA variants, is required for effective precision diagnosis, prognosis, and therapy.

In this paper, we deal with two experimental approaches for the quantitative measurement of *CFTR* mRNA. We compared a relative quantification protocol using real-time PCR (RT-PCR) with an absolute quantification protocol using digital droplet PCR (ddPCR). The ddPCR has not been previously implemented for the general study of the *CFTR* mRNA level. In our knowledge, only a previous study used ddPCR specifically to complete of the molecular diagnosis of two CF patients by studying the *CFTR* mRNA anomalous splicing of a single *CFTR* exon [18].

Our paper is the first comprehensive description of a general ddPCR protocol for *CFTR* mRNA quantification and the first comparison between the ddPCR and RT-PCR methods. The objective was to highlight the features of both methods when applied to *CFTR* mRNA quantification, opening the way to more comprehensive studies about individual levels of *CFTR* gene expression. An enhanced knowledge of the individual *CFTR* mRNA levels, finally affecting CFTR protein levels, would be extremely useful for the more effective application of personalized CF therapies.

## 2. Materials and Methods

**Figure 1:**
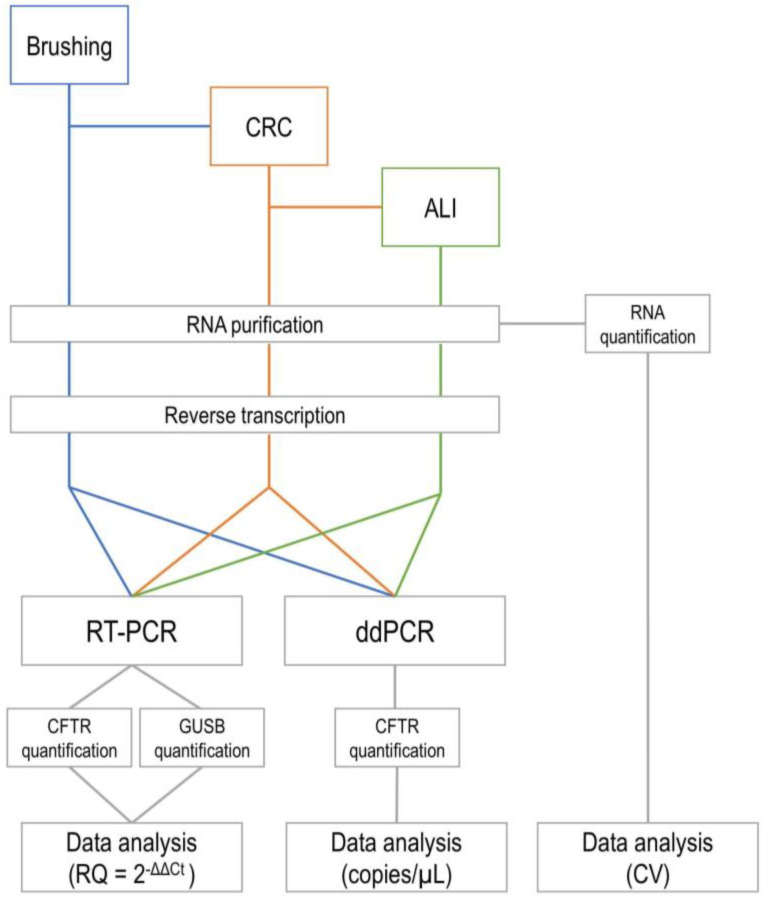
Flowchart of experimental steps for the analysis of the *CFTR* gene expression quantification. The study of analytical variability of RNA dosage after purification is based on the calculation of the coefficient of variation (CV). The *CFTR* expression analysis by RT-PCR is based on threshold cycle and 2^−ΔΔCt^ calculation (using *GUSB* gene as housekeeping gene). The *CFTR* expression analysis by ddPCR is based on the number of positive droplets. See text for explanation. Brushing = nasal brushing specimen; CRC = undifferentiated CF-CRC-AESC; ALI = differentiated CF-CRC-AESC; RQ = relative quantification.

A flowchart of the experimental steps is reported in Figure 1. The experimental procedures are described in detail below.

### 2.1. Case Series, Nasal Brushing and Cell Cultures

Human nasal epithelial samples were collected according to a previously published procedure and case series of individuals who were wild type or affected by CF and enrolled in the CF Reference Center of Lazio Region (Italy) [19]. Briefly, nasal epithelial cells were obtained through cytology brushing (Doctor Brush, AIESI) of the inferior turbinates from both nostrils in DMEM/F12 medium (Gibco) and 5X antibiotics (Penicillin/Streptomycin/Amphotericin B). Recovered cells were cultured as undifferentiated airway epithelial stem-like cells (AESC) by the conditionally reprogrammed cell methodology (CRC) according to our previous protocols [19], consisting of the co-cultivation of epithelial cells with irradiated (30 gray) murine J2 Swiss 3T3 fibroblasts (Kerafast, Boston, MA, USA) in the presence of 10 μM Rock inhibitor Y-27632 (Selleck, Munich, Germany), in F medium (3:1 *v*/*v* F12 Nutrient Mixture Ham (Gibco, Waltham, MA, USA) : DMEM (Gibco)) supplemented with 5% fetal bovine serum (Euroclone, Lima, Peru), 0.4 μg/mL hydrocortisone (Sigma, St. Louis, MO, USA), 5 μg/mL insulin (Sigma), 24 μg/mL adenine (Sigma), 8.4 ng/mL cholera toxin (Sigma), and 10 ng/mL EGF (Peprotech, London, UK). Differentiation was obtained by culturing the CRC cells on transwell inserts (Corning, Glendale, AZ, USA) with the air–liquid interface (ALI) culture conditions to obtain differentiated AESC, as previously described [19,20]. In this paper, 58 experimental samples from the previous case series were used, selected as brushing specimens (N = 19), undifferentiated CF-CRC-AESC (N = 20), and differentiated CF-CRC-AESC (N = 19). All cultures were maintained at 37 °C in a humidified incubator with 5% CO_2_.

### 2.2. DNA Purification, Quantification, and CFTR Mutational Analysis

Genomic DNA was extracted from both the peripheral blood leucocytes of enrolled individuals and derived undifferentiated CF-CRC-AESC, using the QIAamp DNA Blood midi kit (Qiagen, Hilden, Germany) and quantified using fluorimetry (Qubit, Invitrogen, CA, USA). The proximal 5′-flanking, all exons and adjacent intronic zones, and the 3′-UTR of the *CFTR* gene (RefSeq NM_000492.4, NG_016465.4) were sequenced by a Sanger cycle sequencing protocol (ThermoFisher Scientific, Waltham, MA, USA) as previously described [21,22,23], using a genetic analyzer (ABI PRISM 3130*xl*; ThermoFisher Scientific). Genetic analysis was completed through multiplex ligation-dependent probe amplification (SALSA MLPA probemix P091 *CFTR*, MRC Holland, Amsterdam, The Netherlands) to unveil macrodeletions/macroduplications. The genotype of derived undifferentiated CF-CRC-AESC was confirmed using the same methodology. Results of mutational analysis have already been published [5,19].

### 2.3. RNA Purification, Quantification and Reverse Transcription

For both RT-PCR and ddPCR protocols, RNA was extracted from nasal brushing and both undifferentiated and differentiated CF-CRC-AESC using the RNeasy mini kit (Qiagen, Hilden, Germany) and quantified by fluorimetry (Qubit, Invitrogen, CA, USA). Overall, 58 experimental points were collected. Reverse transcription was performed by the iScript cDNA Synthesis kit (Bio-Rad, Hercules, CA, USA), which includes a mix of oligo(dT) and random hexamers as a priming strategy. The following mix was used: 1 μg of total RNA in 5.5 μL, 4 μL of 5X iScript reaction mix, 1 U of iScript reverse transcriptase in 1 μL, and 9.5 μL of H_2_O in a final volume of 20 μL, according to the manufacturer’s instructions. The reactions were incubated in a PTC 100 thermocycler (Bio-Rad) running the following program: 5′ 25 °C, 30′ 42 °C, and 5′ 85 °C.

### 2.4. CFTR Expression Analysis by RT-PCR

The principle of RT-PCR involves the enzymatic amplification of RNA retrotranscribed to cDNA coupled with the addition of specific dyes or fluorescent probes that allow continuous monitoring of amplification in real time. Data analysis involves the use of a threshold cycle. The method usually provides a relative quantification, referred to as a housekeeping gene, which should have a constant average expression in all samples, and to a calibrator experimental condition. Reliability and consistency of results are improved and variations minimized by the use of an analytical reaction triplicate. A recent general description of the RT-PCR method can be found in the paper of Artika and co-workers [24].

**Figure 2:**
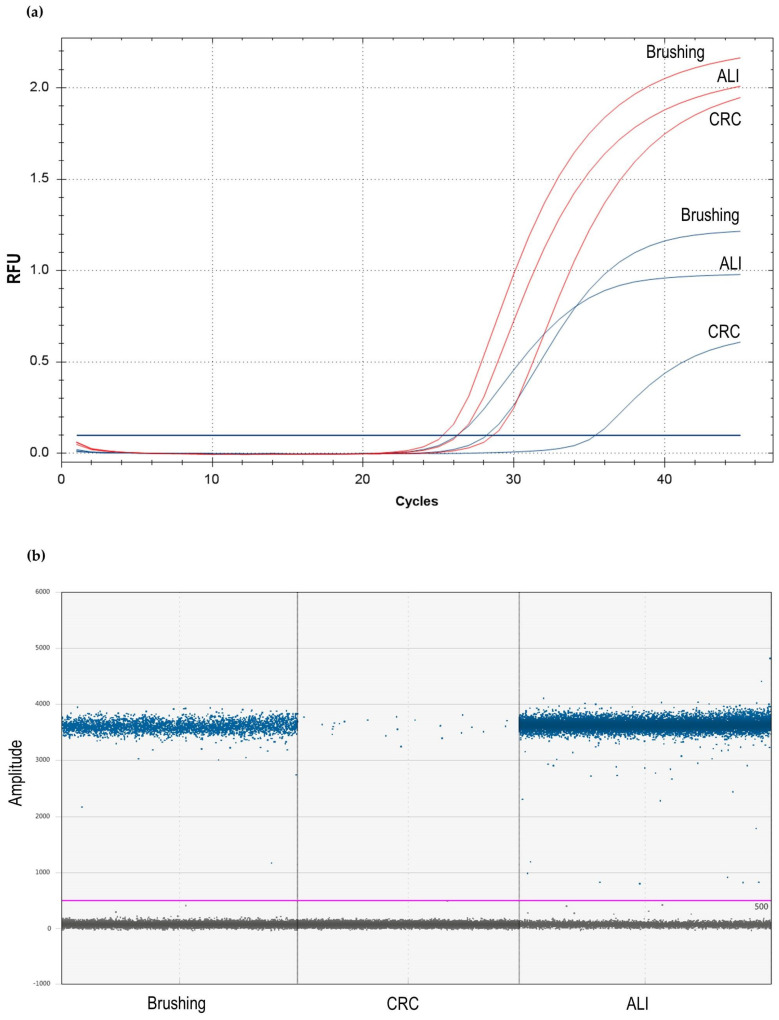
Examples of RT-PCR and ddPCR expression analysis. (**a**) Example of expression analysis relating to one patient’s RT-PCR of *CFTR* gene (blue lines) and *GUSB* gene (red lines). The quantification of gene expression is based on threshold cycle and 2^−ΔΔCt^ calculation (see text for explanation); (**b**) example of expression analysis relating to the same patient of (**a**) in ddPCR of *CFTR* gene. The quantification of gene expression is based on the number of positive droplets (blue dots); gray dots correspond to empty droplets. For both (**a**,**b**): Brushing = nasal brushing specimen; CRC = undifferentiated CF-CRC-AESC; ALI = differentiated CF-CRC-AESC; RFU = relative fluorescence units.

Starting from the cDNA mix described above, a specific no-ROX Master Mix (FluoCycle™ II Master Mix for probe, EuroClone, Milan, Italy) coupled with a TaqMan *CFTR* gene expression assay (code 4331182, ID Hs00357011_m1; ThermoFisher Scientific) or a TaqMan β-glucuronidase (*GUSB*) gene expression assay as reference housekeeping gene (code 4331182, ID Hs00939627_m1) were used, according to the manufacturer’s instructions and our previous protocol [25]. Both probes were FAM dye-labeled. The final reaction volume was 20 μL, using 1 μL of cDNA mix, 10 μL of 2X no-ROX master mix, 1 μL of specific TaqMan probe assay, and 8 μL of H_2_O, according to manufacturer’s instructions. The real-time PCR instrument used was the MJ MiniOpticon (Bio-Rad), with the following program: 5′ 95 °C and 45 cycles of 15″ 95 °C followed by 1′ 60 °C. The threshold cycles of both *CFTR* and *GUSB* genes were acquired in triplicate for each sample. These triplicates were used for the assessment of the analytical variability of the RT-PCR protocol. The analysis was performed using ΔCt, calculated as the difference between the Ct of *CFTR* and the average Ct of *GUSB*, and then calculating the value of 2^−ΔΔCt^, referring to expression results obtained for the lung NL1 cells with wild type *CFTR* [19]. An example of the RT-PCR results is reported in Figure 2a.

### 2.5. CFTR Expression Analysis by ddPCR

In ddPCR, the RNA retrotranscribed to cDNA is amplified into thousands of separate reaction chambers, produced by a water-in-oil emulsion that divides the samples into about 20,000 droplets. PCR amplification occurs in each droplet, and the compartments that contain the target molecule read as positive (through the use of specific dyes or fluorescent probes). Droplets that do not contain the target read as negative. The minimum number of droplets suitable for a reliable assessment of the absolute number of mRNA copies of the gene of interest in ddPCR, giving a Poisson distribution, is around 15,000. The characteristic of ddPCR quantification is to have an absolute count of cDNA molecules (corresponding to mRNA molecules), eliminating the need for calibrator conditions and reference housekeeping genes. A recent general description of the ddPCR method can be found in the paper of Galimberti and co-workers [26].

The ddPCR assays were performed on the same cDNA samples described above and used for RT-PCR. For ddPCR, the final reaction volume was 20 μL, using 1 μL of cDNA mix, 10 μL of the 2X ddPCR Supermix for probes (no dUTP) (Bio-Rad, Hercules, CA, USA, 1863024), 1 μL of the same TaqMan *CFTR* gene expression assay used for RT-PCR (code 4331182, ID Hs00357011_m1; ThermoFisher Scientific), and 8 μL of H_2_O. According to manufacturer’s instructions, 70 μL of droplet generation oil for probes were added. The water-in-oil droplet emulsion was prepared using the QX200 Droplet Generator (Bio-Rad). The amplification step was performed using 40 μL of emulsion in a C1000 thermal cycler (Bio-Rad) using the following program: 10′ 95 °C; 45 cycles of 30″ 94 °C, 1′ 60 °C, followed by 10′ 98 °C. Although common ddPCR protocols do not require analytical triplicates, for the evaluation of the analytical variability of ddPCR and its comparison with that of RT-PCR, we also performed the triplicate for each sample for ddPCR. In our samples, we read a number of droplets within the range from 19,000 to 21,000, well above the required threshold. The ddPCR reactions were analyzed using the QX200 Droplet Reader and QuantaSoft software version 1.7.4 (both from Bio-Rad). An example of the ddPCR results is reported in Figure 2b.

### 2.6. Statistical Analysis

Student’s *t* test, paired Student’s *t* test, regression analysis, and correlation coefficient were used for statistical analysis and assessment of statistical significance using the PRISM software (GraphPad, Boston, MA, USA).

## 3. Results

**Figure 3:**
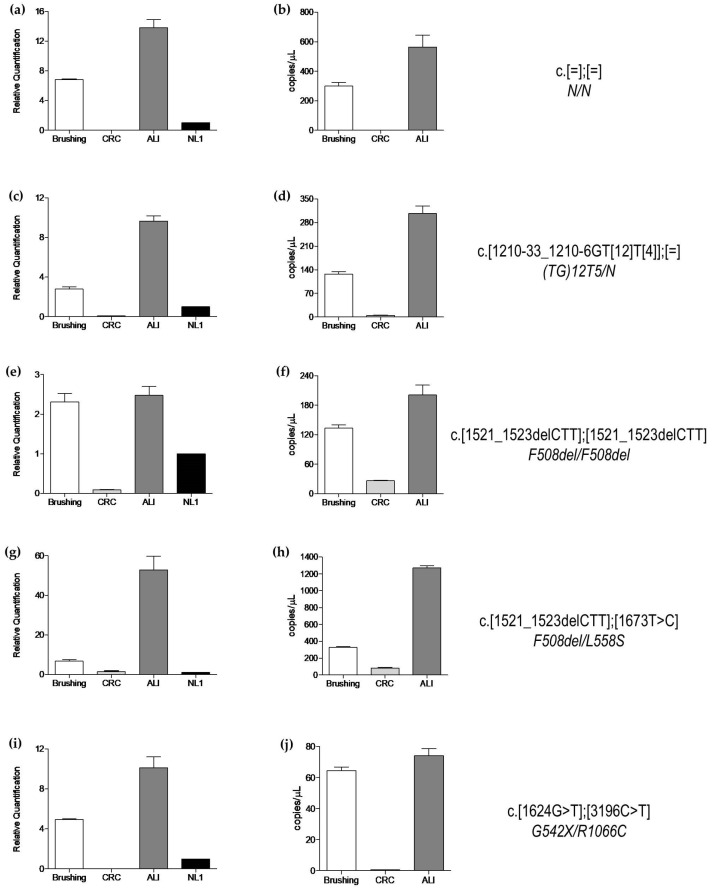
Examples of *CFTR* expression in brushing specimens and CF-CRC-AESC cultures. RT-PCR analysis (**a**,**c**,**e**,**g**,**i**) and ddPCR analysis (**b**,**d**,**f**,**h**,**j**) are reported for the same sample from a single individual with indicated genotype ((**a**,**b**), genotype N/N; (**c**,**d**), genotype (TG)12T5/N; (**e**,**f**), genotype F508del/F508del; (**g**,**h**), genotype F508del/L558S; and (**i**,**j**), genotype G542X/R1066C). The *CFTR* genotypes are indicated on the right (in each panel: upper row, HGVS name; lower row, legacy name). Each bar represents the mean (±S.D.) from biological triplicates. For all panels: Brushing = nasal brushing specimen; CRC = undifferentiated CF-CRC-AESC; ALI = differentiated CF-CRC-AESC; N and [=] = wild type allele. NL1 is the calibrator condition for RT-PCR (see text for explanation).

As previously demonstrated [19], the biological variability of *CFTR* expression in the different experimental conditions used is high. The *CFTR* gene was highly expressed in the brushing samples and differentiated CF-CRC-AESC. On the contrary, in undifferentiated CF-CRC-AESC, the *CFTR* gene expression was very low. Some examples of this variability in the expression of *CFTR* are reported in Figure 3 (only 20 experimental points out of 58 are reported by way of example), where for each individual/genotype, the mean (±S.D.) of biological triplicates is shown.

The analytical variability, related to the quantification of RNA after purification and of *CFTR* expression, is a crucial variable for the reliability of expression results. For this reason, the average coefficient of variation (CV) of the RNA quantification protocol, of the RT-PCR relative quantification of *CFTR* expression, and of the ddPCR absolute quantification of *CFTR* expression were measured.

The RNA quantification method used requires two different protocols depending on the RNA concentration after purification. The high-sensitivity protocol should be used when the RNA concentration is equal to or lower than 10 ng/μL. The broad-range protocol should be used when the RNA concentration is higher than 10 ng/μL, up to 100 ng/μL. Accordingly, the average CVs of the RNA quantification using the high-sensitivity and broad-range protocols were calculated as 12.4% ± 7.5 (N = 11) and 1.3% ± 0.6 (N = 32), respectively (Table 1, rows of the RNA purification protocol). These differences are statistically significant (Student’s *t* test, *p* = 0.0007).

Similarly, the overall average CVs, as well as the CV for experimental conditions with low (undifferentiated CF-CRC-AESC) and high (brushing and differentiated CF-CRC-AESC) *CFTR* quantification, were calculated for both RT-PCR and ddPCR. For the RT-PCR, the overall CV was 11.2% ± 7.0. The conditions with low *CFTR* expression corresponded to a RQ ≤ 1 (which means that *CFTR* expression was equal to or less than the calibrator experimental condition). Five outliers (with RQ ≤ 0.07 and very variable results) were excluded from the analysis. The CVs of the RT-PCR for the low and high *CFTR*-expressing conditions were 14.9% ± 8.8 (N = 15) and 9.8% ± 5.7 (N = 38), respectively (Table 1, rows of RT-PCR protocol), which were statistically significantly different (Student’s *t* test, *p* = 0.02). For the ddPCR, the overall CV was 9.0% ± 7.4. The difference in the overall CV between the ddPCR and the RT-PCR protocols was statistically significant (paired Student’s *t* test, *p* = 0.03). The conditions with low *CFTR* expression corresponded to a number of *CFTR* copies/μL ≤ 26. In this case, three outliers (common to the RT-PCR protocol, with copies/μL ≤ 1 and very variable results) were excluded from the analysis. The CVs of ddPCR for the low and high *CFTR*-expressing conditions were, respectively, 12.8% ± 8.7 (N = 17) and 7.3% ± 6.3 (N = 38) (Table 1, rows of ddPCR protocol), which were statistically significantly different (Student’s *t* test, *p* = 0.01). The differences between the RT-PCR and ddPCR results for low and high *CFTR*-expressing conditions were not statistically significant.

**Figure 4:**
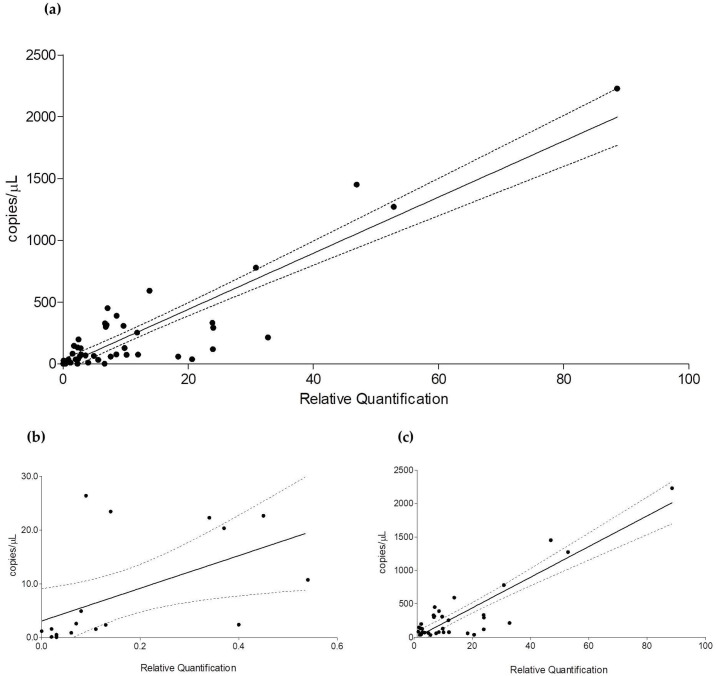
Correlation between results of *CFTR* quantification obtained using RT-PCR and ddPCR. (**a**) Correlation for any value of *CFTR* expression (N = 58; R^2^ = 0.8201, *p* < 0.0001); (**b**) correlation for low *CFTR*-expressing conditions (N = 20; R^2^ = 0.2817, *p* < 0.05); (**c**) correlation for high *CFTR*-expressing conditions (N = 38: R^2^ = 0.7915, *p* < 0.0001). RT-PCR results are shown on x-axis (as relative quantification); ddPCR results are shown on y-axis (as absolute quantification). Solid line represents the regression line; dotted lines represent the 95% confidence interval. Some experimental points could not be visible for overlap.

Taking advantage from the highly differentiated levels of expression and low analytical variability, a correlation analysis between the *CFTR* gene expression measured using RT-PCR and that measured using ddPCR was performed. As for the correlation analysis we used the average values, it appeared suitable not to exclude the experimental points we defined as outliers due to high analytical variability. In fact, their average values (although low) could be determined by both methods. As shown in Figure 4a (taking into account 58 experimental points), there is an excellent correlation between the two methodological approaches (regression analysis: R^2^ = 0.8201, *p* < 0.0001). The correlation is maintained also for low (regression analysis: R^2^ = 0.2817, *p* < 0.05) (Figure 4b) and high (regression analysis: R^2^ = 0.7915, *p* < 0.0001) (Figure 4c) *CFTR*-expressing conditions.

## 4. Discussion

It is well known that the *CFTR* gene is highly expressed in respiratory epithelia. In keeping with this, it is well expressed in nasal epithelia, as in brushing specimens. In the patient-specific cellular models we setup [19], when nasal epithelial cells were reverted to a stem-like phenotype as undifferentiated CF-CRC-AESC, *CFTR* expression became very low. When differentiation is re-induced by ALI culture conditions, *CFTR* expression raised, reaching, in some cases, expression levels higher than in brushing. In some cases, the effect of the mutated genotypes added further variability to the physiological interindividual variability, enhancing the differences in *CFTR* gene expression between the different experimental samples analyzed.

The analytical variability of the RNA quantification protocol appeared to be greater when the RNA concentration was low. In our quantification protocol, when the RNA concentration is equal to or lower than 10 ng/μL, the average CV is 12.4%, as compared to a CV of 1.3% when the RNA concentration is higher than 10 ng/μL (a difference that is statistically significant). Although even the value of 12.4% imprecision introduced by the RNA quantification is expected to have a minimal impact on the subsequent quantification of *CFTR* expression, it is advisable to obtain an RNA concentration higher than 10 ng/μL to reach the minimum imprecision level of 1.3%.

Taking advantage of the use, for each sample, of the same specimen of purified RNA, retrotranscription mix, and TaqMan assay for both protocols, the analytical variability of *CFTR* quantification by RT-PCR and ddPCR was assessed and compared. The overall analytical variability, measured by the total *CFTR* expression, was lower for ddPCR (CV = 9.0%) than for RT-PCR (CV = 11.2%) (a statistically significant difference). Considering the variable level of *CFTR* expression, the analytical variability of both the RT-PCR and ddPCR protocols was higher when *CFTR* expression was low. For RT-PCR, the CV is 14.9% if RQ ≤ 1, with respect to a CV of 9.8% when RQ > 1 (a statistically significant difference). For ddPCR, the CV is 12.8% if the number of copies/μL ≤ 26, with respect to a CV of 7.3% if the number of copies/μL > 26 (a statistically significant difference). The ddPCR had lower imprecision than the RT-PCR for any level of *CFTR* expression, although the reduced sample size due to the stratification of sampling between low and high *CFTR*-expressing conditions prevented the achievement of statistical significance (achieved, on the contrary, by the analysis of the overall CVs in unstratified sampling, as reported above).

Additional effects of the imprecision of the RNA and *CFTR* quantification are expected. In the worst experimental conditions (low concentration of RNA and low expression of *CFTR*), the CVs are 12.4% (RNA quantification) and 14.9% (RT-PCR quantification of *CFTR* expression) and 12.4% (RNA quantification) and 12.8% (ddPCR quantification of *CFTR* expression). In these conditions, the loss of precision can be ascribed at a similar level to the RNA quantification and to the *CFTR* expression quantification. In the best experimental conditions (high concentration of RNA and high expression of *CFTR*), the CVs are 1.3% (RNA quantification) and 9.8% (RT-PCR quantification of *CFTR* expression) and 1.3% (RNA quantification) and 7.3% (ddPCR quantification of *CFTR* expression). In these conditions, the greatest loss of precision can be ascribed to the *CFTR* expression quantification. In both conditions, the overall imprecision was lower for the ddPCR-based procedure than for the RT-PCR-based procedure.

Although ddPCR has been used in CF for diagnostic purposes [27] or for the characterization of *CFTR* splicing variants [18], to our knowledge, this is the first published paper analyzing *CFTR* expression by a comprehensive ddPCR method and also comparing it with a RT-PCR approach. Due to the excellent correlation between the measurements of *CFTR* expression using RT-PCR and ddPCR, both methods appeared to be well suited for the quantification of *CFTR* gene expression. However, ddPCR has some advantages over RT-PCR. One of the advantages is its greater precision in the evaluation of both a low and high level of *CFTR* expression. Another advantage of ddPCR is its ability to achieve an absolute quantification of the mRNA/cDNA copies of the *CFTR* gene without the need to use a housekeeping reference gene and a calibrator experimental condition. Finally, the fact that ddPCRs do not need analytical triplicates for each specimen should not be neglected. In addition, the high analytical sensitivity of ddPCR in several applications is well recognized [28,29,30,31], although we did not specifically address this in this paper.

Several sources of variability are involved in the translation from *CFTR* mRNA to mature CFTR protein. Among these, the mutated genotype can affect the protein production. For example, a discrepancy between the *CFTR* mRNA and protein levels could be found in CF individuals with pathogenic variants that affect the protein’s maturation and function. However, in other cases, it is conceivable that the final CFTR protein level could be proportional to the mRNA quantity, for example, when the *CFTR* mRNA is low, as in non-sense-mediated decay due to stop codons. Additionally, interindividual variability can originate from the differentiated levels of *CFTR* mRNA. These are still unclear points that deserve further study. In effect, better knowledge of the correlation between the levels of *CFTR* mRNA and protein should be mandatory for diagnostic, prognostic, and therapeutic issues. At least in some cases, it could be possible that the *CFTR* mRNA copies measured using ddPCR may be predictive of protein expression.

The highest analytical performances, such as those of ddPCR, are often required for a *CFTR* expression evaluation. For example, in some genotypes involving splicing variants with a variable phenotypical effect [32], it appears to be mandatory to base functional interpretation and clinical decisions more on the residual *CFTR* function than on genotype. In these cases, the CFTR residual function mainly depends on the amount of *CFTR* wild-type mRNA, and its exact measurement appears mandatory. More generally, although with the limitations about the correlation between mRNA and protein level discussed above, for the correct application of personalized modulatory therapies, the quantification of wild-type *CFTR* mRNA may constitute a useful tool for precision diagnostics on which to base precision therapeutic options.

## 5. Conclusions

Both RT-PCR and ddPCR appeared to be suitable approaches for *CFTR* expression quantification. The ddPCR approach showed enhanced characteristics with respect to RT-PCR, such as the possibility to quantify *CFTR* expression in terms of the absolute number of mRNA/cDNA *CFTR* copies without the need for a reference gene or a calibrator experimental condition, with greater precision and a lower number of analytical replicates. The enhanced methods for *CFTR* gene expression quantification represent useful tools for a more complete understanding of *CFTR* expression patterns, a prerequisite for precision diagnostics and prognostics. In turn, in the era of precision medicine for CF, the accurate assessment of individual *CFTR* levels of expression may have important consequences for personalized therapeutic approaches. Future research aimed at the clarification of biological interindividual variability in *CFTR* expression appears to be required.

## Figures and Tables

**Table 1:** Average coefficient of variation (CV) of RNA dosage after purification and of *CFTR* quantification by RT-PCR and ddPCR protocols. See footnote and text for statistical analysis and significance.

Protocol	Level of RNA Concentration or *CFTR* Expression	Average Coefficient of Variation of the RNA or *CFTR* Quantification (CV% ± S.D.)
RNA purification	RNA concentration ≤ 10 ng/μL (N = 11)	12.4 ± 7.5
	RNA concentration > 10 ng/μL (N = 32)	1.3 ± 0.6
RT-PCR	Total *CFTR* expression (N = 53)	11.2 ± 7.0
	Low *CFTR* expression (RQ ≤ 1; N = 15) (undifferentiated CF-CRC-AESC)	14.9 ± 8.8
	High *CFTR* expression (RQ > 1; N = 38) (brushing and differentiated CF-CRC-AESC)	9.8 ± 5.7
ddPCR	Total *CFTR* expression (N = 55)	9.0 ± 7.4
	Low *CFTR* expression (copies/μL ≤ 26; N = 17) (undifferentiated CF-CRC-AESC)	12.8 ± 8.7
	High *CFTR* expression (copies/μL > 26; N = 38) (brushing and differentiated CF-CRC-AESC)	7.3 ± 6.3

Footnote on statistical significance about CV comparisons: RNA purification; RNA concentration ≤ 10 ng/μL vs. RNA concentration > 10 ng/μL, Student’s *t* test *p* = 0.0007 (significant)—total *CFTR* expression; RT-PCR vs. ddPCR, paired Student’s *t* test *p* = 0.03 (significant)—RT-PCR; low *CFTR* expression vs. high *CFTR* expression, Student’s *t* test *p* = 0.02 (significant)—ddPCR; low *CFTR* expression vs. high *CFTR* expression, Student’s *t* test *p* = 0.01 (significant)—low *CFTR* expression; RT-PCR vs. ddPCR, paired Student’s *t* test not significant (*p* = 0.1)—high *CFTR* expression; RT-PCR vs. ddPCR, paired Student’s *t* test not significant (*p* = 0.2).

## Data Availability

All the relevant data have been provided in the manuscript and any dataset used and/or analyzed during the current study are available from the corresponding author on reasonable request.
